# Efficacy of the Coronoid Notch and Occlusal Plane As Predictable Landmarks for Determining the Medial Cut Height in Bilateral Sagittal Split Osteotomy: A Split-Mouth Observational Study

**DOI:** 10.7759/cureus.70812

**Published:** 2024-10-04

**Authors:** Venkata Saikrishna Yalagala, Shanmugasundaram Somasundaram, Krishnakumar Raja, Bharan Ravindran, John Rozar Raj

**Affiliations:** 1 Department of Oral and Maxillofacial Surgery, Sri Ramaswamy Memorial (SRM) Dental College and Hospital, Ramapuram, IND

**Keywords:** coronoid, lateral bone cut ends, mandibular foramen, orthognathic, split

## Abstract

Aim: This split-mouth observational study was conducted to assess the reliability and safety of using the coronoid notch and occlusal plane as landmarks to aid surgeons during bilateral sagittal split osteotomy (BSSO).

Materials and methods: Thirteen patients between the ages of 18 and 30 years, with class II and class III mandibular skeletal malocclusion requiring BSSO, were randomly selected and assigned to each of the study and control groups. A split-mouth study was chosen to conduct this research. Cone beam computed tomography (CBCT) imaging was conducted before the surgery to evaluate the anatomical structure, and three predefined points were marked at the superiormost point of the mandibular foramen, the inferiormost point of the mandibular foramen, and the deepest point of the sigmoid notch. A conventional sagittal split osteotomy was carried out in the control group. Preoperative values were obtained in the study group using the CBCT imaging technique by drawing an imaginary line from the inferiormost part of the mandibular foramen to the line corresponding to the occlusal plane that extended beyond the last molar. The lingual flap reflection was restricted to the internal oblique ridge. The posterior border of the mandible was not reflected. The measurements acquired via CBCT imaging were accurately transferred to the intraoperative surgical site using a vernier caliper. This facilitated the precise completion of the horizontal osteotomy, ascending ramus cuts, and vertical osteotomies. BSSO was performed, the mandibular setback or advancement was done with intermaxillary fixation, and the procedure was completed by rigid fixation. Types of lingual splits, types of lateral bone cut ends (LBCEs), any unfavorable split, and the time taken for the surgery (in minutes) were assessed.

Results: The surgery time in the control group (20.1538 ± 2.85325 min) was found to be higher than that in the study group (17.6154 ± 3.59487 min), with a p-value of 0.02. No significant differences were observed when assessing the presence of unfavorable splits in both groups (p = 0.500). Buccal LBCEs were the most prevalent, followed by inferior types. Type I lingual split was the most common in the study group (70%).

Conclusion: This technique offers a dependable anatomical reference and significantly reduces surgical time for beginners. Additionally, the patterns of the lingual split were correlated with the types of lateral bone cut ends.

## Introduction

In oral and maxillofacial surgery, optimal outcomes depend on the accuracy of surgical techniques. When performing procedures such as bilateral sagittal split osteotomy (BSSO), it is crucial to identify reliable anatomical landmarks that can accurately guide surgical interventions [1]. The coronoid notch is a crucial feature for determining the height of the medial cut in BSSO [2]. The aim of this study was to determine the reliability of the coronoid notch and occlusal plane as guiding landmarks, especially for beginners in the field. This study provides essential insights that can improve the precision and effectiveness of BSSO techniques by investigating the reliability and uniformity of this anatomical feature [3]. The use of the coronoid notch and occlusal plane as a guiding landmark promises to simplify and refine the surgical approach, providing a practical and reliable tool for surgeons entering the intricate domain of oral and maxillofacial surgery [4,5].

A BSSO is a milestone in the history of orthognathic surgery. In 1907, Blair described the horizontal subcondylar osteotomy of the mandible to treat class II dysgnathia by advancement of the mandibular body. Schuchardt's method changed the horizontal flat osteotomy by introducing a method that carried out cortical osteotomies obliquely, starting from just above the lingula and reaching the buccal cortex 1 cm further downward without contacting the intra-alveolar nerve (IAN). Trauner and Obwegeser further refined Schuchardt's method and increased the distance between the horizontal incisions to 25 mm. Chiseling along the lateral cortex eventually resulted in the fracture of the ramus. The buccal cortex of the mandibular body between the first and second molars was formed by the advancement and rotation of the lower horizontal cut, which was a result of Dal Pont's modification. The angle created between the lingual and buccal cortical cuts was approximately 90°, leading to an extension of the connecting cut along the oblique line on the lateral mandibular aspect and across the mylohyoid groove on the lingual side. A possible strategy to consider is the lingual short split modification, which may have a higher possibility of paresthesia recovery and minimize the manipulation of the inferior alveolar neurovascular bundle [6].

The lingual, sagittal, and horizontal osteotomies are modified to allow for a downward osteotomy when the chisel and Smith retractor are positioned. By positioning the horizontal osteotomy beneath the lingula's entry, the insertion of the pterygomasseteric muscle has been maintained, but the cortical area seems to have weakened. Due to improved temporomandibular joint (TMJ) stability after surgery and reduced risks of inferior alveolar neurovascular bundle trauma and retromandibular blood vessel bleeding, less mucoperiosteal detachment of the mandibular ramus lingual surface can minimize surgical trauma and the likelihood of complications [7].

BSSO is a well-established surgical procedure commonly used in oral and maxillofacial surgery to correct a variety of dentofacial deformities. A crucial stage within this intricate technique is the medial cut, a key maneuver involving the sagittal separation of the mandibular ramus. Determination of the optimal height for a medial cut is a nuanced decision that necessitates a comprehensive understanding of the facial anatomy, surgical skill, and a meticulous approach. To achieve functional and cosmetic harmony, the medial cut in BSSO is crucial in reshaping the mandible and aligning maxillary and mandibular arches. The height of the medial cut profoundly influences postoperative stability, occlusal outcomes, and patient satisfaction. Therefore, exploring factors that determine the selection of medial cut height is a critical focus in oral and maxillofacial surgery. The foundation for a thorough analysis of the complexities surrounding the medial cut in BSSO is laid forth next. As we embark on a journey to unravel the complexities of this essential surgical step, the objective is to contribute to the collective understanding of the nuances involved. This will help advance the knowledge base of practitioners engaged in the challenging landscape of oral and maxillofacial surgery [8].

Precise and successful procedures are contingent upon the accurate identification of anatomical landmarks. BSSO, a cornerstone in the correction of dentofacial deformities, demands meticulous planning and execution. Among the various anatomical references available, the coronoid notch has emerged as a potential and intriguing landmark for determining the height of the medial cut in BSSO. The aim of this research is to determine the reliability of the coronoid notch and occlusal plane as landmarks for guiding surgeons, especially those who are new to the field. The selection of an appropriate medial cut height is crucial for achieving optimal surgical outcomes in BSSO procedures. The coronoid notch, which is located at the anterior border of the mandibular ramus, is a distinctive feature that can be used as a reliable guide in this context. As the effectiveness of this anatomical landmark in assisting surgeons remains an area warranting investigation, this study seeks to contribute valuable insights into its predictability and consistency [8].

One crucial aspect of performing BSSO lies in determining the optimal height of the medial cut, a factor that significantly influences the surgical outcome. This study explores a new and potentially reliable anatomical landmark to help beginners establish the appropriate height for the medial cut in BSSO. Through a split-mouth design, this study seeks to evaluate the efficacy of providing a predictable and consistent reference point for surgeons entering the intricate domain of BSSO. By investigating this specific landmark, the study aims to contribute valuable insights to the evolving field of oral and maxillofacial surgery, offering a practical approach for beginners to enhance precision and efficacy in performing BSSO procedures.

## Materials and methods

Patients with skeletal malocclusion were selected from the Department of Oral and Maxillofacial Surgery, Sri Ramaswamy Memorial (SRM) Dental College and Hospital, Ramapuram, Chennai, for this prospective randomized controlled clinical study. The study was carried out between March 2022 and March 2024. Patients aged 18-30 years with skeletal class II or class III malocclusion, as confirmed by cephalometric analysis, were included in the study. This criterion ensured precision and clarity in diagnosing specified malocclusions in the study population. In contrast, patients aged 30 years and above, those with medical comorbidities, and individuals with any anatomical variation in the dentition were excluded from the study. The SRM Dental College and Hospital Institutional Review Board gave ethical approval (SRMDC/IRB/2021/MDS/No. 403). Bilingual informed consent was acquired from the individuals who were selected according to the inclusion criteria. They were informed of the goals and research procedures and reassured that their participation in the study was voluntary and that they might discontinue at any time. They were also told that the information would be strictly confidential and used only for legitimate scientific research.

Under the supervision of the head of the Oral and Maxillofacial Surgery department at the SRM Dental College and Hospital, an investigator was appropriately trained and calibrated to carry out the procedures. Both interventions were administered by a single calibrated researcher, who also recorded all performances. Thirteen patients were included in the split-mouth study; two samples (from the right and left sides) from each patient were collected using the split-mouth technique of every patient and separated into two groups: 13 in the study group and 13 in the control group. Of the 13 study patients, seven underwent surgery on the right side and six on the left, with the contralateral side serving as the control group. A detailed medical and dental history was recorded, and clinical examination and cone beam computed tomography (CBCT) imaging were performed to determine the alignment and structure of the jaw.

**Figure 1:**
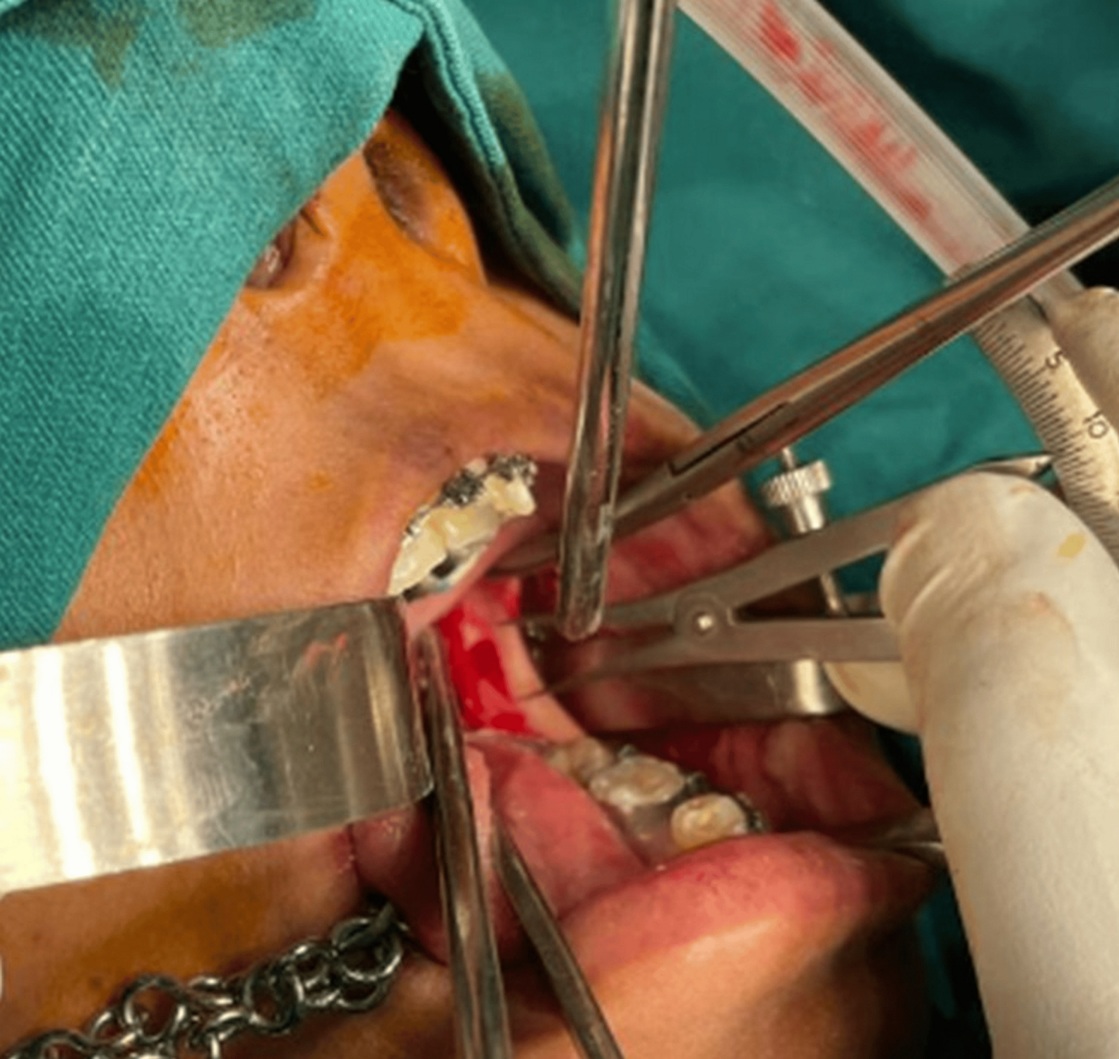
Sagittal osteotomy performed on a patient in the study group

The primary outcome measure was evaluating the predictability of the coronoid notch and short sagittal split osteotomy techniques in orthognathic surgeries. The secondary outcome measure was assessing the preoperative horizontal and vertical distances, time taken for the surgery, any unfavorable splits, types of lateral bone cut ends (LBCEs), and lingual splits. Figure 1 shows sagittal osteotomy performed for one of the patients in the study group.

**Figure 2:**
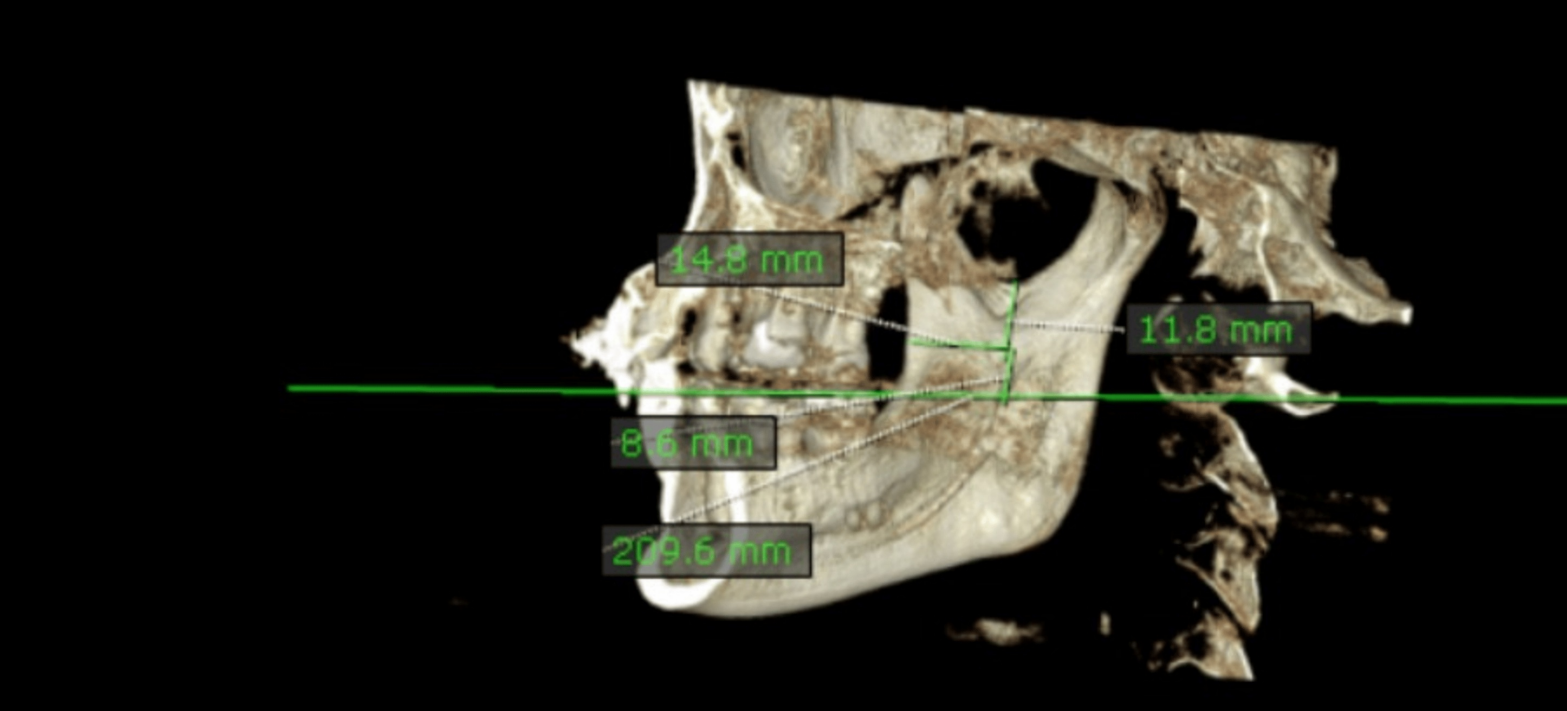
Preoperative measurements taken for the study group

A defined surgical protocol was used to perform the surgery. A single surgeon carried out all surgeries on each patient under general anesthesia. In the study group, an incision was made from 34 to the ascending ramus of the mandible region, and a full-thickness mucoperiosteal flap was elevated. Reference marking was done according to the preoperative CBCT assessment values. Preoperative CBCT was done for all the patients. Three predefined points were marked, and measurements were taken (Figure 2).

A horizontal line was drawn from the superiormost point of the mandibular foramen, extending perpendicular to the long axis of the ascending ramus. A vertical line was drawn from the inferiormost point of the mandibular foramen to the line corresponding to the occlusal plane extending beyond the last molar. A vertical line was drawn from the sigmoid notch to the superiormost point of the mandibular foramen. The patient was placed in a supine position on the operating table with general nasotracheal intubation, and was prepared and draped for an intraoral procedure. Bilateral inferior alveolar nerve blocks with a short-acting local anesthetic and vasoconstrictor were given, which was supplemented by a long-acting anesthetic at the end of the procedure.

**Figure 3:**
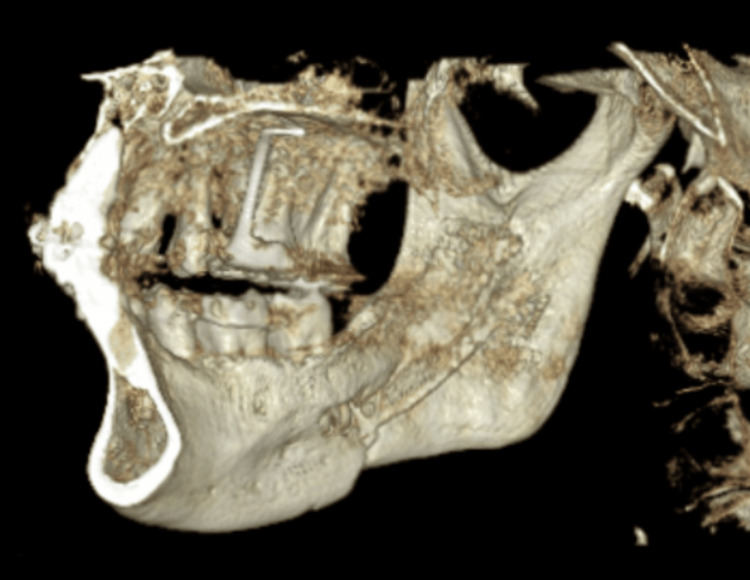
A postoperative day-7 image for one of the patients in the study group

The classical and conventional third molar incision and osteotomy were done in the control group. In the study group, preoperative values were obtained from CBCT images by drawing an imaginary line from the inferiormost part of the mandibular foramen to the line corresponding to the occlusal plane extending beyond the last molar. The reflection of the lingual flap was restricted to the internal oblique ridge. The posterior border of the mandible was not reflected. A vernier caliper was used to transfer the values obtained from the CBCT images to the intraoperative surgical site, where the horizontal osteotomy, ascending ramus, and vertical cuts were completed. The BSSO was performed, and the mandibular setback or advancement was done with intermaxillary fixation (IMF) using a surgical wafer. The procedure was completed by rigid fixation. Osteotomies were placed horizontally on the medial surface of the ramus, following the sagittal osteotomy and the vertical osteotomy distal to the first molar. Mandibular setbacks and advancements were done with surgical splints fixed with IMF. Fixation was done, and IMF was released. The occlusion and dental midline were checked and closure was done with 3-0 vicryl sutures. Figure 3 shows the postoperative day 7 image for one of the patients in the study group. The preoperative and postoperative day 7 CBCT images were compared and analyzed.

The parameters were evaluated with the following scores for lingual splits: type I represented the fracture line running as a vertical fracture line to the inferior mandibular border, type II represented the fracture line running with the medial bone cut above the lingula and extending toward the posterior border, type III represented the fracture line running through the mandibular foramen and obliquely extending toward the mandibular angle, type IV represented the fracture line running through the mandibular canal to the inferior border of the mandible, type V represented the fracture with the medial bone cut extending through the mandibular foramen toward the posterior border of the ramus and the mandibular remaining in the distal fragment, and type VI represented other unexpected fractures. The assessment of an unfavorable split was marked as Yes, scored as 1, and No, scored as 0. Assessments of lateral bone cut ends (lingual, inferior, or buccal) and time taken (in minutes) were also recorded.

The results of the Kolmogorov-Smirnov and Shapiro-Wilk tests (normality tests) revealed that the variables were normally distributed. Therefore, parametric and nonparametric methods were applied to analyze the data. Independent samples were compared using an unpaired t-test. Categorical variables were compared with the groups using the chi-square test. SPSS Statistics for Windows, version 26.0 (IBM Corp., Armonk, NY) was used to analyze the data. The significance level was set at p = 0.05.

## Results

Table 1 displays the data regarding the time taken for the surgical procedures in both groups. A statistically significant difference was found between the groups. The study group took less time than the control group, with a p value of 0.02.

**Table 1:** Time taken for the surgery N: number of patients

Group statistics	Group	N	Mean	Standard deviation	p value
Time taken for the surgical procedure (in minutes)	Study group	13	17.6154	3.59487	0.02
	Control group	13	20.1538	2.85325	

Table 2 shows the postoperative assessment of unfavorable splits in the study and control groups. Only one patient out of 13 in the study group had unfavorable splits, but no significant differences were noted between the groups.

**Table 2:** Postoperative assessment of unfavorable splits in the study and control groups

Assessments			Group		Total	p value
			Study group	Control group		
Postoperative assessment of unfavorable splits	No	Count	12	13	25	0.500
		% within the postoperative assessment of unfavorable splits	48%	52%	100%	
	Yes	Count	1	0	1	
		% within the postoperative assessment of unfavorable splits	100%	0	100%	
Total		Count	13	13	26	
		% within the postoperative assessment of unfavorable splits	50%	50%	100%	

Table 3 shows the postoperative assessment of lateral bone cut ends in the study and control groups. The buccal bone cut was observed in 80% of the participants in the study group, with a p value of 0.026, followed by an inferior bone cut. Statistically significant differences were observed in the buccal cut bone ends in the study group.

**Table 3:** Postoperative assessment of lateral bone cut ends in the study and control groups

Assessments			Group		Total	p value
			Study group	Control group		
Postoperative assessment of lateral bone cut ends	Buccal	Count	8	2	10	0.026
		% within the postoperative assessment of lateral bone cut ends	80%	20%	100%	
	Inferior	Count	5	8	13	
		% within the postoperative assessment of lateral bone cut ends	38.5%	61.5%	100%	
	Lingual	Count	0	3	3	
		% within the postoperative assessment of lateral bone cut ends	0	100%	100%	
Total		Count	13	13	26	
		% within the postoperative assessment of lateral bone cut ends	50%	50%	100%	

Table 4 shows the postoperative assessment of the lingual split in the study and control groups. A significant difference was noted in the type I lingual split, followed by type III, with a p value of 0.03, in the study group.

**Table 4:** Postoperative assessment of the lingual split in the study and control groups

Assessments			Group		Total	p value
			Study group	Control group		
Postoperative assessment of the lingual split	Type I	Count	7	3	10	0.03
		% within the postoperative assessment of the lingual split	70%	30%	100%	
	Type II	Count	0	3	3	
		% within the postoperative assessment of the lingual split		100%	100%	
	Type III	Count	5	5	10	
		% within the postoperative assessment of the lingual split	50%	50%	100%	
	Type IV	Count	1	1	2	
		% within the postoperative assessment of the lingual split	50%	50%	100%	
	Type V	Count	0	1	1	
		% within the postoperative assessment of the lingual split	0	100%	100%	
Total		Count	13	13	26	
		% within the postoperative assessment of the lingual split	50%	50%	100%	

## Discussion

BSSO is a common, dependable, and adaptable surgical technique used for correcting mandibular abnormalities, such as prognathism and retrognathism, and facial asymmetries [9]. The following three osteotomies were performed during this surgical procedure: horizontal osteotomy (medial) performed through the lingual cortex above the mandibular foramen, followed by a connecting vertical osteotomy through the anterior side of the ramus, and a lateral osteotomy through the buccal side of the mandibular body. The proximal and distal portions of the mandible were separated manually in a sagittal fashion once the osteotomy was finished, and they were then further adjusted as intended. Despite its established efficacy and safety, problematic side effects such as unfavorable fractures and IAN injuries have been found [10,11]. These clinical difficulties emphasize how crucial it is to manage bone split following BSSO to minimize the danger of IAN damage and unexpected fractures while also assisting in achieving the desired result. This split-mouth observational study was conducted with 13 patients who required a BSSO. A classical third molar incision and osteotomy were performed for the control and study groups. To prevent soft-tissue manipulation, the flap reflection was maintained at a minimum and did not extend to the posterior border of the mandible in the study group.

The primary goal of the study was to compare the aforementioned technique with the conventional one and determine its effect on the manipulation of soft tissues. Our study assessed three preoperative measurements. The first was obtained by drawing a horizontal line perpendicular to the superiormost point of the mandibular foramen, extending to the anterior part of the ascending ramus. The second was obtained by drawing a vertical line from the inferiormost point of the mandibular foramen to the line corresponding to the occlusal plane extending beyond the last molar, and the third was obtained by drawing a vertical line from the sigmoid notch to the superiormost point of the mandibular foramen. As a surgical point during the surgery in the study group, we maintained a distance of at least 3 mm above the mandibular foramen, as per the predetermined values. This was to prevent the damage to the nerve passing through the foramen.

Zhou et al. found that 84.3% of the mandibular foramina were situated 4.5 mm below the occlusal plane. Furthermore, the mean distance of the mandibular lingula from the occlusal plane was 5.9 mm above the plane [12]. The duration of the surgery was noted for both groups (Table 1). The mean value (17.6 ± 3.5 min) for the study group was substantially lower than that of the control group, with a p value of 0.02. The applied techniques required less time, enhanced precision, and produced better results. From our experience, we noted that surgeons took less time to perform when they gained expertise with modified short split osteotomies. When evaluating unfavorable splits after surgery, 48% of the study group and 52% of the control group showed no unfavorable splits (Table 2). Out of 13, one patient in the study group showed unfavorable splits, but no statistically significant differences were noted. This outcome was consistent with the research by Kriwalsky et al. who found that 6% of fractures were unfavorable. BSSO frequently resulted in an undesired fracture of the jaw at the proximal or distal portion, referred to as a "bad split" [13]. Unfavorable splits could cause pseudoarthrosis, infections, delayed bone healing, and bony sequestration of the pieces [13]. It was also possible to have postoperative instability, recurrence, or mandibular dysfunction coupled with TMJ impairment [14]. However, no such complications were observed in our study groups. Our study also assessed lateral bone cut ends (Table 3). Lingual, inferior, and buccal lateral bone cuts might occur after bilateral sagittal osteotomies. According to the classification, the bone cut ends of the lingual type extended across the inferior border of the jaw, the bone cut ends of the inferior type ended at the inferior border of the mandible, and the bone cut ends of the buccal type ended at the buccal surface. The lateral buccal bone cut ends were present in 80% of the study group. With a p value of 0.026, statistically significant differences were seen among the buccal lateral bone cut end, the inferior lateral bone cut end, and the lingual lateral bone cut end. It could be argued that, in line with earlier accounts, this could be because of an incomplete osteotomy at the inferior border of the mandibular body [15]. Furthermore, some research asserted that the buccal plate was strengthened, and the likelihood of a poor split was reduced in the lingual form of LBCE, which included the entire thickness of the bottom border. However, lower border abnormalities might develop in the osteotomy gap due to this condition, particularly in patients who had substantial mandibular advancement [16-18].

Plooij et al. [19] examined the length and location of the medial bone cut during a horizontal osteotomy and found that extending the medial bone cut through the mandibular canal might lower the chance of splitting. Hunsuck's description of the likelihood of splitting was supported when the bone cut end was behind the mandibular foramen.

A total of 30 patients met the criteria described by Hunsuck. Among them, 15 experienced buccal fractures, while the remaining 33 were classified into five distinct categories based on their lingual split patterns. The position of the lateral bone incision performed during the vertical osteotomy, which was done on the buccal side in every incidence of buccal fracture, was the primary factor influencing this split propensity [16]. Muto et al. reported that mandibular prognathism is relatively common among Asian populations [18]. Some researchers proposed techniques for separating the inferior border and emphasized the importance of conserving the lingual cortex of the inferior border with the distal piece. These techniques involved the use of saws with specialized designs or ultrasonic equipment. These investigations unequivocally showed that a complete osteotomy with an inferior LBCE at the inferior border and lingual cortex preservation improved the lingual split and avoided an undesired fracture [15]. Many studies found an association between LBCE and neurosensory disturbances, but our study found no significant association between them [15]. Hu et al. concluded that there was a significant association between LBCE and split types [15]. Their study stated that buccal bone cut ends tended to have type V and VI lingual split types, but our study had lateral buccal bone cut ends and type I lingual split. A postoperative assessment of the lingual split was also done in our study (Table 4), and statistically significant differences were observed (p = 0.03). Compared with the study group, conventional osteotomies had a higher incidence of lingual split types. Type I lingual split was observed more frequently (70%) than type III and IV splits in the study group. The anatomical characteristics of the mandibular ramus affected the split patterns during BSSO. According to Mensink et al., this kind of split was "good" rather than "bad," particularly when there was asymmetry or a transverse cant, and the posterior aspect of the ramus was free of bony obstruction [17]. Muto et al., on the other hand, discovered that a lateral bone cut that crossed the mandibular inferior border and a short lingual cut made just above the lingula resulted in an appropriate lingual split pattern as well. The coronoid notch was used as a reliable perceptible marker to conduct BSSO on the study group [18].

We used the coronoid notch as a predictable and palpable landmark to perform the BSSO in the study group. According to Kim et al. [20], in 82.0% of patients, the level of the coronoid notch's deepest point coincided with the tip of the lingula. According to Fujimura et al. and Kositbowornchai et al., the shape of the lingulae affected the location of the lingula and the mandibular foramen. The locations changed in accordance with the varying shape, and there was a possibility of damaging the inferior alveolar nerve due to his mutation [21-23]. It was found that inadequate anesthesia during the inferior alveolar nerve block was associated with structural abnormalities in the lingula and incorrect localization of the mandibular foramen [24-27].

The nerve injury was minimal when there was a space of more than 3 mm between the lingula and the mandibular foramen [12]. Many factors, such as the surgeon's competence, fixation procedures, age at the time of surgery, gender, IAN exposure, and intraoperative time, were found to be substantially linked to the incidence of IAN injuries after BSSO. Based on our research, we found that CBCT assessment before performing the osteotomy procedure and planning the horizontal cut at a minimum distance of 3 mm above the mandibular foramen, along with minimal soft-tissue exposure, resulted in minimal injury to the IAN and prevented bad splits. This procedure required less surgical time, even for young surgeons, and helped with precise buccal cuts, thus reducing bad splits.

It is important to note that this study has certain limitations. In order to accurately estimate the dimensions of the mandible and identify anatomical characteristics in relation to stable landmarks, such as the coronoid notch or the occlusion plane, larger sample sizes involving specific dentofacial deformities, such as class II or class III, are required. The split-mouth design used in the study may result in potential biases due to differences in anatomical structures between the study and control groups of each patient. In order to gain a more comprehensive understanding of the safety and effectiveness of using the coronoid notch and occlusal plane as reference points in BSSO procedures, future research should incorporate a broader range of outcome measures and utilize randomized controlled trials with larger sample sizes.

## Conclusions

Bilateral sagittal osteotomy, performed with minimal soft-tissue exposure without flap reflection until the posterior border, reduces tissue damage. Our study confirms that utilizing the coronoid notch as a landmark and performing the horizontal osteotomy cut at a level 3 mm above the mandibular foramen effectively prevent inferior alveolar nerve damage during surgical procedures. This technique offers a reliable, predictable, and safer anatomical landmark, with the advantage of reduced surgical time for beginners. Patterns of buccal bone cuts were more significant in the study group, indicating a higher prevalence and better precision in achieving desired osteotomy outcomes. The clear delineation of buccal bone cuts also suggests that this method can lead to improved surgical accuracy and reduced intraoperative complications, thereby enhancing overall surgical efficiency and patient safety.
